# High-dose methotrexate-based regimens and post-remission consolidation for treatment of newly diagnosed primary CNS lymphoma: meta-analysis of clinical trials

**DOI:** 10.1038/s41598-020-80724-0

**Published:** 2021-01-22

**Authors:** Junyao Yu, Huaping Du, Xueshi Ye, Lifei Zhang, Haowen Xiao

**Affiliations:** Department of Hematology, Sir Run Run Shaw Hospital, Zhejiang University School of Medicine, No. 3 Qingchun East Rd., Hangzhou, 310016 Zhejiang Province People’s Republic of China

**Keywords:** Cancer, Diseases, Medical research, Neurology, Oncology

## Abstract

With the exception of high-dose methotrexate (HD-MTX), there is currently no defined standard treatment for newly diagnosed primary central nervous system lymphoma (PCNSL). This review focused on first-line induction and consolidation treatment of PCNSL and aimed to determine the optimal combination of HD-MTX and the long-term beneficial consolidation methods. A comprehensive literature search of MEDLINE identified 1407 studies, among which 31 studies met the inclusion criteria. The meta-analysis was performed by using Stata SE version 15. Forest plots were generated to report combined outcomes like the complete response rate (CRR), overall survival, and progression-free survival. We also conducted univariate regression analyses of the baseline characteristics to identify the source of heterogeneity. Pooled analysis showed a CRR of 41% across all HD-MTX-based regimens, and three- and four-drug regimens had better CRRs than HD-MTX monotherapy. In all combinations based on HD-MTX, the HD-MTX + procarbazine + vincristine (MPV) regimen showed pooled CRRs of 63% and 58% with and without rituximab, respectively, followed by the rituximab + HD-MTX + temozolomide regimen, which showed a pooled CRR of 60%. Pooled PFS and OS showed that post-remission consolidation with autologous stem cell transplantation (ASCT) was associated with the best survival outcome, with a pooled 2-year OS of 80%, a 2-year PFS of 74%, a 5-year OS of 77%, and a 5-year PFS of 63%. Next, whole-brain radiation therapy (WBRT) + chemotherapy showed a pooled 2-year OS of 72%, 2-year PFS of 56%, 5-year OS of 55%, and 5-year PFS of 41%, with no detectable CR heterogeneity throughout the entire treatment process. In HD-MTX-based therapy of newly diagnosed PCNSL, MPV with or without rituximab can be chosen as the inductive regimen, and the rituximab + HD-MTX + temozolomide regimen is also a practical choice. Based on our study, high-dose chemotherapy supported by ASCT is an efficacious approach for consolidation. Consolidation with WBRT + chemotherapy can be another feasible approach.

## Introduction

Primary central nervous system lymphoma (PCNSL) is an uncommon type of extranodal non-Hodgkin lymphoma (NHL) that occurs in the central nervous system (CNS), such as in the brain parenchyma, meninx, medulla spinalis, or eyes without any evidence of systemic disease outside the CNS. It accounts for 4% of primary intracranial tumors and 4–6% of extranodal NHL in immunocompetent populations^1^.

The most common histopathological type of PCNSL is CD20-positive diffuse large B cell lymphomas (DLBCL). Due to the blood–brain barrier (BBB), the treatment of PCNSL typically differs from that of peripheral lymphomas. The classic CHOP regimen proved ineffective in the treatment of PCNSL^2–4^.

High-dose methotrexate (3.5–8 g/m^2^) can reportedly transit through the BBB. In the 1970s, HD-MTX monotherapy was recognized as effective in treating PCNSL with overall response rates (ORRs) of 35–74%, a median progression-free survival (PFS) of 10–12.8 months, and a median overall survival (OS) of 25–55 months^5–7^. Further studies confirmed a significant improvement in tumor responses and survival outcomes when other chemotherapeutic agents were added to HD-MTX^8,9^.

PCNSL is also sensitive to radiotherapy. Whole-brain radiation therapy (WBRT) was initially applied as the only treatment^10^, whereas in recent years, after several reports on the efficacy of HD-MTX-based chemotherapy, WBRT has been more widely applied as part of a combination consolidation treatment^9,11^. Alternatively, high-dose chemotherapy supported by autologous stem cell transplantation (HDC/ASCT) is another therapy for this disease as post-remission consolidation^12,13^. A broad consensus exists in the field that PCNSL should be treated with optimal induction chemotherapy based on systemic HD-MTX followed by the appropriate consolidation therapy^14^.

In this article, we first systematically summarized the efficiency of each chemotherapy combination with a backbone of HD-MTX as the first-line induction treatment and then evaluated the survival outcomes of the different methods in the following consolidation stage.

## Methods

### Literature search

We performed a comprehensive English literature search in the PubMed database published through August 2019. We searched for all clinical trials that focused on first-line treatment of immunocompetent newly diagnosed PCNSL patients. Two investigators independently reviewed potentially eligible titles, abstracts, and full texts.

### Inclusion and exclusion criteria

Studies were included according to the following criteria: (1) randomized controlled trials (RCT) or single-arm clinical trials in phase 2 or higher; (2) studies using HD-MTX as a single agent or in combination with other drugs as first-line treatment; and (3) studies that had clearly documented treatment outcomes of CRR, PFS, and OS. We excluded: (1) reviews, case reports, and animal research; (2) retrospective studies; (3) studies of systematic lymphoma involving the CNS; and (4) studies that excluded elderly or younger patients.

### Investigated treatments

The general approach of the initial treatment of PCNSL is high dose methotrexate. In our study, we tried to determine whether the combination of other chemical agents with HD-MTX will have better outcomes versus mono HD-MTX, and which combination will be superior in terms of a complete response. First, we categorized each therapy as the number of components: (1) one agent (mono regimen), (2) two agents (dual regimen), (3) three agents (triplet regimen), (4) four agents (tetrad regimen), and (5) five or more agents (multiple regimen). Then, we compared the regimens with different combinations based on HD-MTX: (1) HD-MTX (M), (2) HD-MTX + Ara-C, (3) HD-MTX + procarbazine + vincristine (MPV), (4) rituximab (R) + HD-MTX + temozolomide (TMZ), (5) HD-MTX + carmustine + procarbazine + vincristine (MBPV), (6) rituximab + HD-MTX + procarbazine + vincristine (R-MPV), and (7) HD-MTX + Ara-C + rituximab + thiotepa (thio). We further explored the effects of different post-remission consolidation therapies on the PCNSL patients and comparatively analysed the overall and progression-free survival outcomes of the following four consolidation regimens: (1) WBRT, (2) WBRT + chemical agents, (3) ASCT, and (4) chemical agents only.

### Statistical analysis

All statistical analyses were performed using Stata SE, version 15 (StataCorp, College Station, Texas, USA). We used the Cochrane Q statistic (*P* < 0.1 considered significant) and I^2^ index (25–50% representing low, 50–70% moderate, and > 75% high heterogeneity) to calculate the degree of heterogeneity of the studies. A random-effects model was used if there was significant heterogeneity (*P* < 0.1 or I^2^ > 50%). Otherwise, a fixed-effects model was employed. Forest plots were generated to report the results, wherein the sample volume was shown by the size of the square and the 95% confidence interval by the lines on each side of the square.

Because of the substantial heterogeneity and the lack of randomization, meta-regression was used to determine the independent effect of the following variables on the heterogeneity across studies: gender (male percentage), median age, performance status including Karnofsky Performance Status (KPS) scale (KPS median), Eastern Cooperative Oncology Group (ECOG) scale (percentage of ECOG ≤ 1), mono or multiple lesion sites (multiple sites percentage), histopathological type (diffuse large B cell lymphoma (DLBCL) percentage), positive for CSF cytology and protein (percentage), the involvement of the ocular system (percentage), MTX dose and the number of cycles in the induction treatment. The meta-regression analysis was performed with Stata SE using the “metareg” command with random-effects models. A *P* value of < 0.05 was used to define the covariates that were significantly correlated with the uncontrollable sources of heterogeneity. A univariate regression analysis was performed first, and as no more than one covariate was significant, a multivariate model was not applied.

## Results

### Literature search

A literature search in PubMed identified 1407 studies related to the treatment of PCNSL. After restricting the search to clinical trials, 152 studies remained, of which 121 studies were excluded for one of the following reasons: irrelevant topics, focus on elderly patients, relapsed or refractory diseases, duplicate, insufficient data, not in the English language, review articles, and editorials. Finally, according to our inclusion criteria, we identified 31 eligible studies published between 1992 and 2019, including two subsequent study reports of the same clinical trial (Ferreri^8^ and Ferreri^15^).

### Study characteristics

We extracted the following baseline characteristics from each study: author, year, number of total and male patients, median age, performance status, brain lesion sites, histopathological type, cerebrospinal fluid (CSF) and ocular involvement, induction and consolidation treatment regimens, and efficacy outcomes.

The induction therapy of the included studies had a backbone of HD-MTX, combined with other chemical drugs, such as cytarabine, ifosfamide, temozolomide, procarbazine, vincristine, and rituximab. The outcome CRR after chemo-induction treatment was utilized for further analysis. In the consolidation stage, we focused on the post-remission regimens of WBRT, ASCT, chemotherapy, chemotherapy combined with WBRT, and their survival outcomes of 2-year and 5-year OS and PFS. The baseline characteristics of each study are summarized in Table 1.

**Table 1:** Characteristics of the included studies.

References	No	Male	Age, median	KPS, median	EGOG ≤ 1	Multiple sites	DLBCL	CSF cytology	CSF protein	Ocular	Induction	Consolidation	CR best^d^	Single dose of MTX (mg/m^2^)	The number of Cycles^e^
**Single-arm clinical trials**															
Abrey^17^	52	37	65	70	NR	NR	NR	34	NR	10	MPV	WBRT + chemo	45	3.5	5
Abrey^18^	28	18	53	70	NR	NR	NR	3	NR	3	MTX + AraC	ASCT	8	3.5	5
Adhikari^19^	22	13	51.5	NR	4	15/20	18	3/22	19/21	5/22	RMPV	WBRT + chemo	10	3.5	5
Batchelor^5^	25	17	59.8	78.4 (mean)	NR	14/24	22	3/14	NR	5	MTX	chemo	12	8	6
DeAngelis^20^	31	16	5	NR	NR	NR	14	5	NR	2	MTX	WBRT + chemo	27	1	2
DeAngelis^21^	98	53	56.5	80	NR	NR	NR	17/81	NR	NR	MPV	WBRT + chemo	29	2.5	5
Ferreri^16^	13	6	54	NR	10	6	NR	1	NR	2	MPV	WBRT	9	3	4.6
Ferreri^22^^a^	41	–	–	–	–	–	–	–	–	–	MTX + AraC + thio + IDA	WBRT	23	3.5	2.4
Glass^23^	25	16	NR	60	NR	16	25	9	NR	5	MTX	WBRT	22	3.5	3.2
Glass^24^	53	25	57	NR	42	NR	48	NR	NR	NR	R + MTX + TMZ	WBRT + chemo	18	3.5	5
Herrlinger^25^	37		60	70	NR	14	NR	4/27		3/28	MTX	chemo	11	8	6
Illerhaus^26^	30	25	54	70	NR	8	NR	NR	NR	NR	MTX + AraC + thio	ASCT	21	8	3
Illerhaus^13^	79	56	44	90	NR	54	77	NR	NR	2	R + MTX + AraC + thio	ASCT	61	8	4
Korfel^27^	56		60	NR	24	NR	52	13/45	NR	NR	BMPD	WBRT + chemo	34	1.5	3
Montemurro^28^	23	12	55	70	NR	12	NR	NR	11/15		MTX	ASCT	17	8	2
Morris^29^	52	30	60	70	NR	NR	NR	11	NR	5	RMPV	WBRT + chemo	34	3.5	6
Omuro^12^	32	17	57	80	NR	NR	32	3/31	NR	3	RMPV	ASCT	21	3.5	6.2
O’Brien^30^	46	33	58	NR	22	19	NR	3/42	NR	2/45	MTX	WBRT	32	1	NR
Pels^31^	65	34	62	70	NR	NR	65^b^	7	NR	1	Multiple^c^	chemo	37	5	6
Poortmans^32^	52	35	51	70	NR	21	NR	7/33	NR	2/51	MBVP	WBRT	36	3	4
Rubinstein^33^	44	21	61	NR	36	NR	43	10	20	1	R + MTX + TMZ	chemo	29	8	4
Wu^11^	25	16	57	NR	10	22	NR	NR	15	NR	MTX + AraC	WBRT	10	3.5	4
**Randomized controlled trials (RCTs)**															
Bromberg^34^	100	61	61	NR	71	51	99	16	NR	3	MBVP	WBRT + chemo	66	3	4
	99	48	61	NR	73	46	97	18	NR	8	RMBVP	WBRT + chemo	67	3	4
Ferreri^9^	40	-	58	NR	20	25	35	2/35	11/35	5	MTX	WBRT	11	3.5	4
	39	-	59	NR	25	21	34	3/34	16/34	4	MTX + AraC	WBRT	25	3.5	4
Ferreri^15^	59	41	58	NR	48	31	NR	NR	42	1	R ± MTX + AraC ± thio	WBRT	55	3.5	4
	59	30	58	NR	39	34	NR	NR	35	3	R ± MTX + AraC ± thio	ASCT	56	3.5	4
Thiel^35^	318	183	61	80	NR	NR	273	158	93	165	MTX + IFO	WBRT	56/154	4	6
	318	183	61	80	NR	NR	273	158	93	165	MTX + IFO	chemo	96/164	4	6

*MTX (M)* methotrexate, *AraC* cytarabine, *IDA* idarubicine, *IFO* ifosfamide, *B* carmustine (BCNU), *P* procarbazine, *V* vincristine, *R* rituximab, *TMZ* temozolomide, *thio* thiotepa, *WBRT* whole-brain radiotherapy, *ASCT* autologous stem cell transplantation, *chemo* chemotherapy, *NR* not reported.

^a^Patients’ characteristics information is not available in the supplementary material.

^b^B-cell origin.

^c^Including high-dose methotrexate, cytarabine, vinca-alkaloids, ifosfamide, and cyclophosphamide.

^d^The best CR number mentioned in the study; some are CR patients after inductive chemotherapy; some are after the completion of the consolidation treatment.

^e^The number of cycles given in inductive treatment which containing MTX.

### Regimens with different numbers of agents

**Figure 1:**
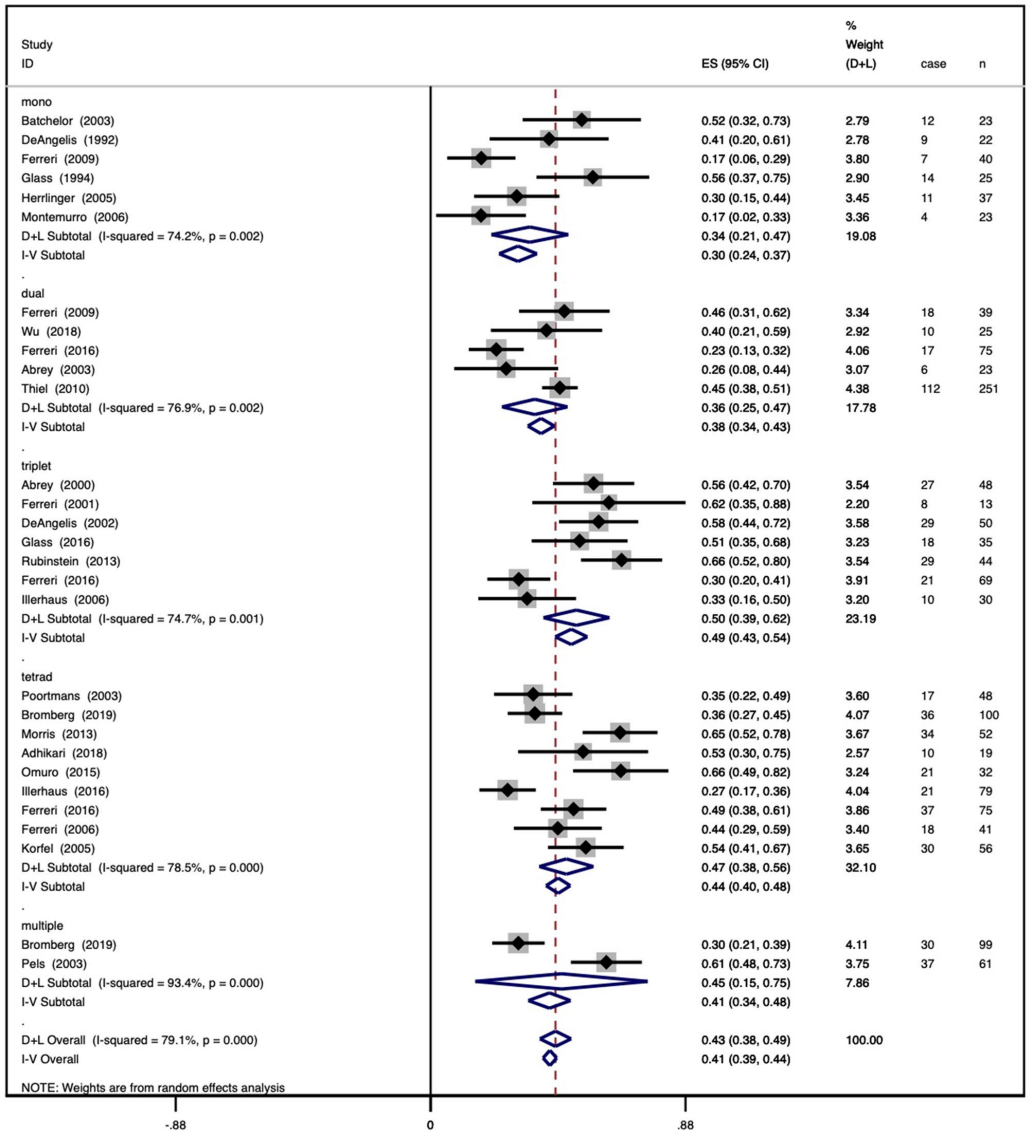

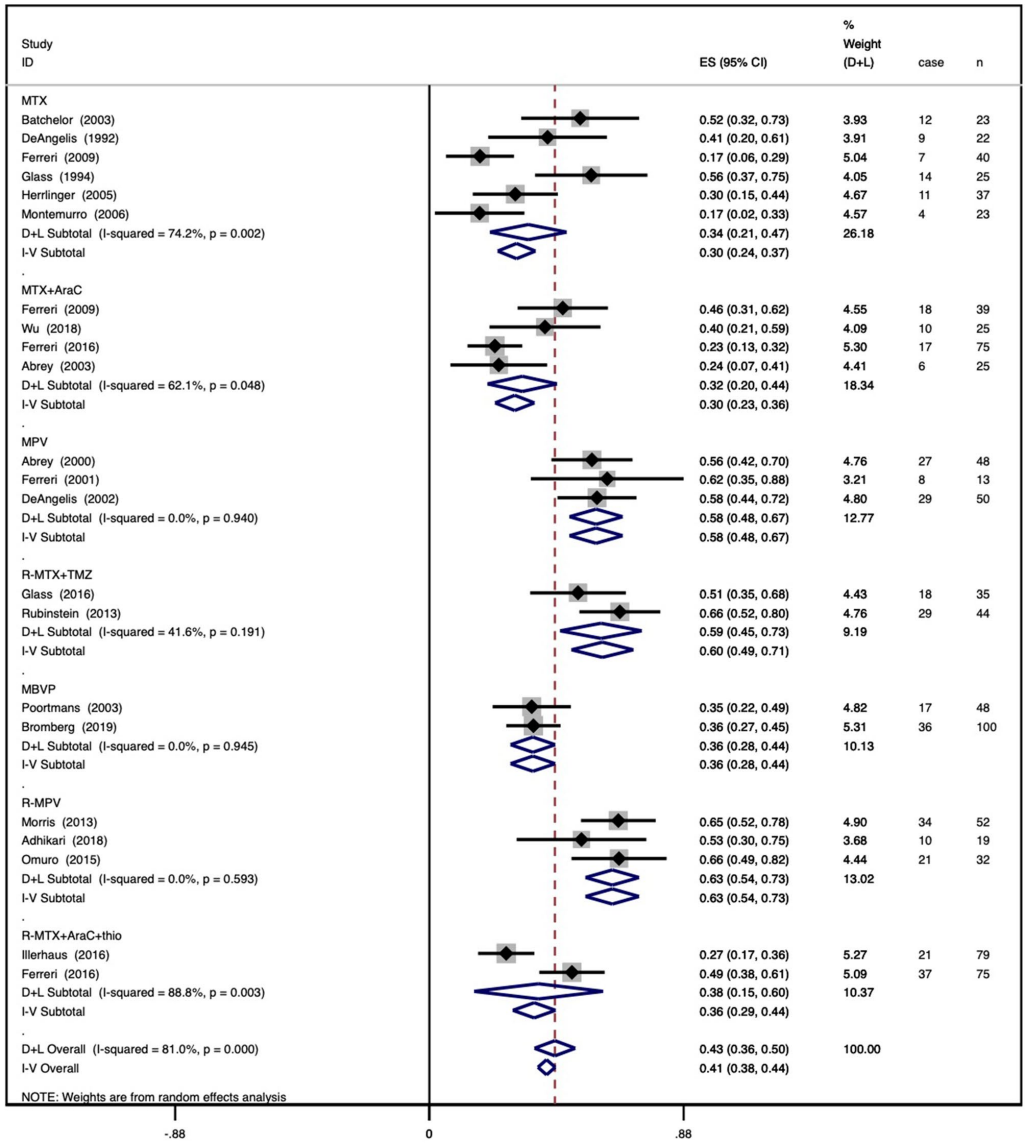
Forest plot of complete remission for regimens. (**a**) Regimens with different numbers of agents and (**b**) regimens with different chemotherapy combinations. A square and bar represents the effect size and 95% CI for each study, respectively, and the size of the square reflects each study’s relative weight. The diamond represents the pooled CRR and 95% CI.

The pooled CRR for six studies with a mono regimen was 0.30 (95% CI 0.24–0.37), with an I^2^ value of 74.2%. Five studies with a dual regimen included a total of 413 patients, with a pooled CRR of 0.38 (95% CI 0.34–0.43) and an I^2^ value of 76.9%. The pooled CRR for seven studies with a triplet regimen was 0.49 (95% CI 0.43–0.54) with an I^2^ value of 74.7%. The pooled CRR for nine studies with a tetrad regimen was 0.44 (95% CI 0.40–0.48), with an I^2^ value of 78.5%. The pooled CRR for two studies with a multiple regimen was 0.41 (95% CI 0.34–0.48), with an I^2^ value of 93.4%. All of the results are shown as a forest plot in Fig. 1a.

### Regimens with different chemotherapy combinations

The pooled CRR for six studies in the MTX group was 0.30 (95% CI 0.24–0.37), with an I^2^ value of 74.2%. The pooled CRR for four studies in the MTX + AraC group was 0.30 (95% CI 0.23–0.36) with an I^2^ value of 62.1%. The pooled CRR for three studies in the MPV group was 0.58 (95% CI 0.48–0.67), with an I^2^ value of 0.0%. The pooled CRR for two studies in the R-MTX + TMZ group was 0.60 (95% CI 0.49–0.71), with an I^2^ value of 41.6%. The pooled CRR for two studies in the MBVP group was 0.36 (95% CI 0.28–0.44), with an I^2^ value of 0.0%. The pooled CRR for three studies in the R-MPV group was 0.63 (95% CI 0.54–0.73), with an I^2^ value of 0.0%. The pooled CRR for two studies in the R-MTX + AraC + thio group was 0.36 (95% CI 0.29–0.44), with an I^2^ value of 88.8%. A forest plot of the results is shown in Fig. 1b.

### OS and PFS for disparate consolidation methods

**Figure 2:**
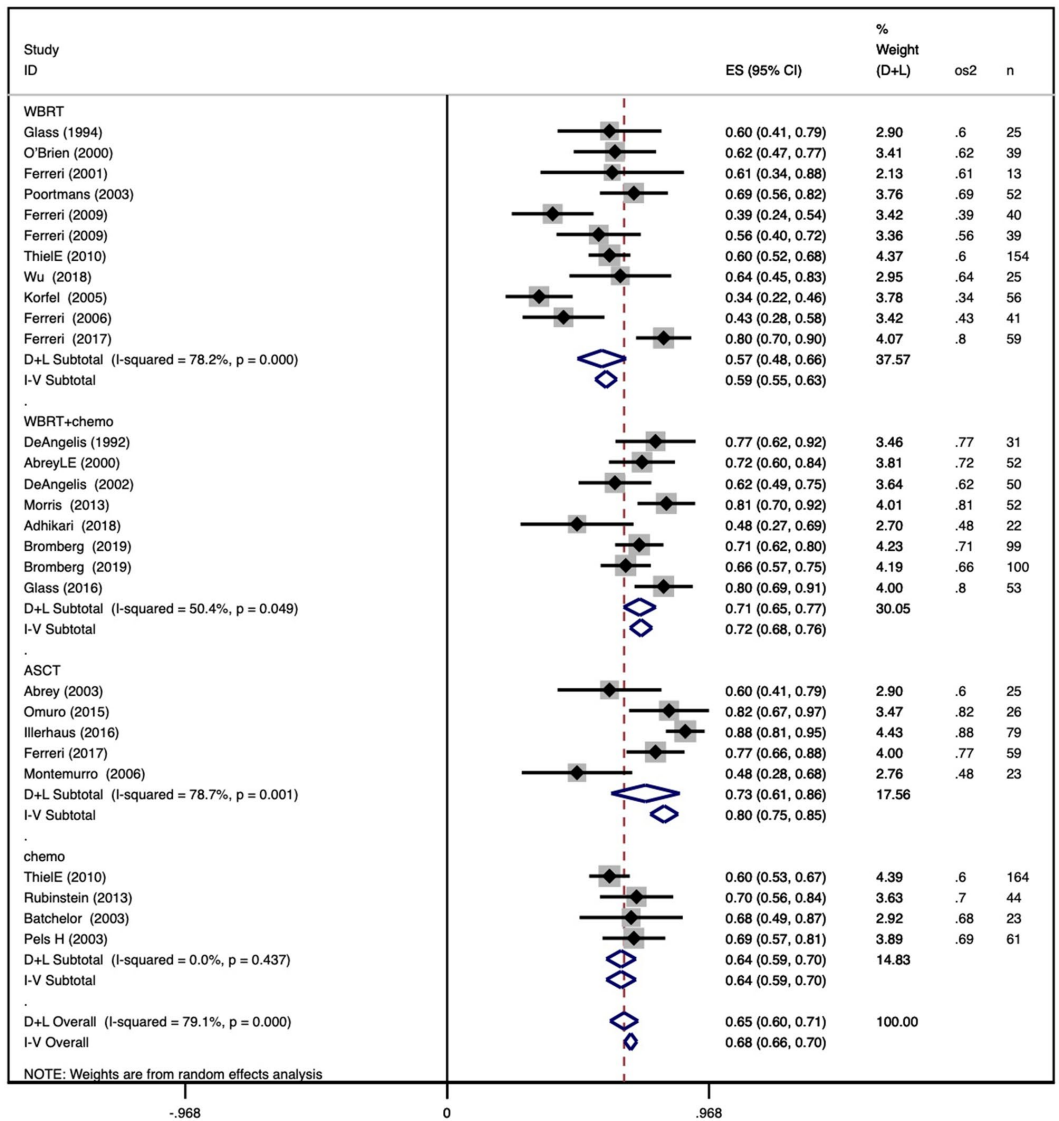

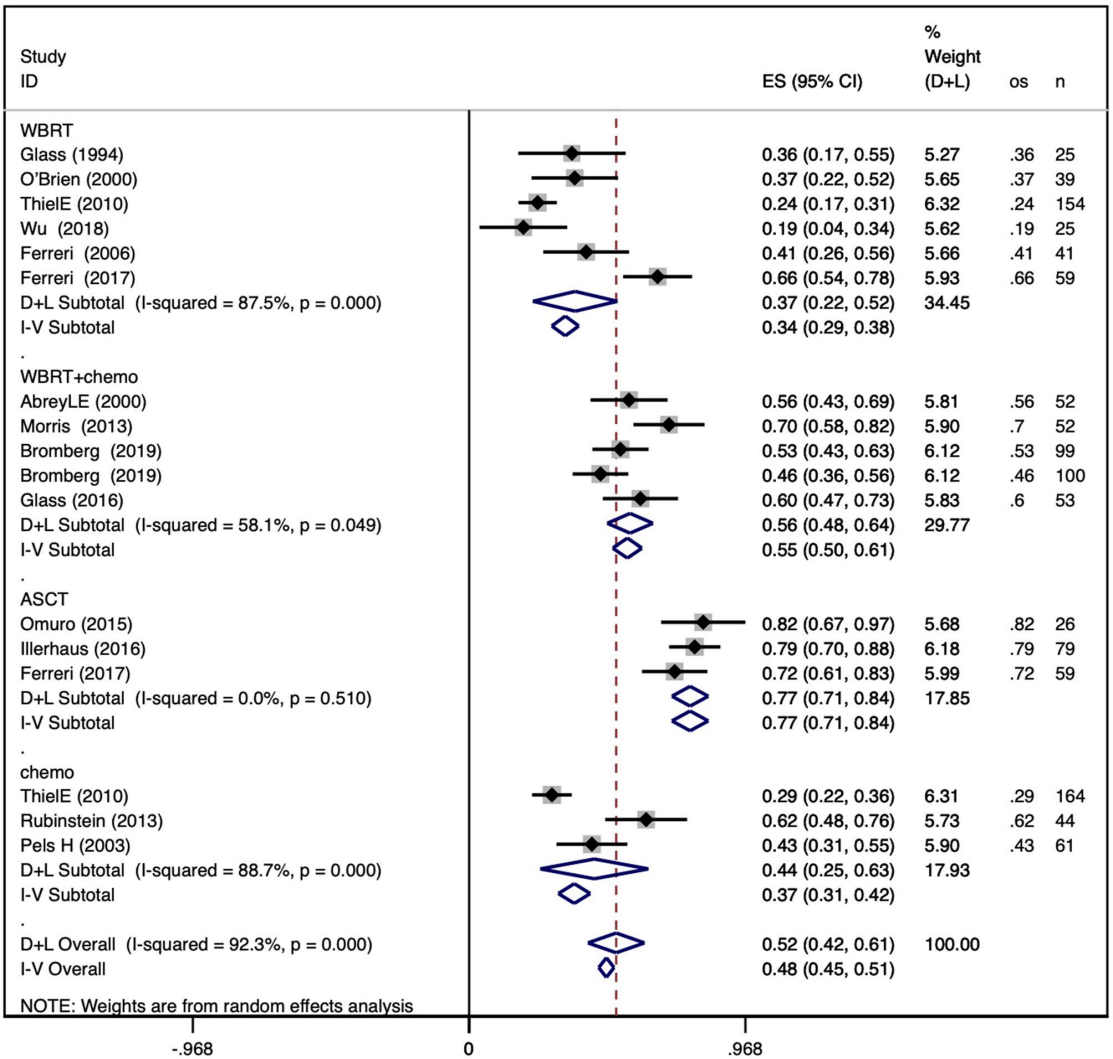
Forest plot of 2-year and 5-year overall survival within different consolidation methods. (**a**) Pooled 2-year overall survival for WBRT, WBRT + chemotherapy, ASCT, and chemotherapy-only consolidation; (**b**) pooled 5-year overall survival for the four consolidation methods.

The pooled 2-year OS in the WBRT group was 0.59 (95% CI 0.55–0.63), with an I^2^ value of 78.2%. The pooled 2-year OS in the WBRT + chemotherapy group was 0.72 (95% CI 0.68–0.76), with an I^2^ value of 50.4%. The pooled 2-year OS in the ASCT group was 0.80 (95% CI 0.75–0.85), with an I^2^ value of 78.7%. The pooled 2-year OS in the chemotherapy group was 0.64 (95% CI 0.59–0.70), with an I^2^ value of 0.0%. The results are shown in Fig. 2a.

The pooled 5-year OS in the WBRT group was 0.34 (95% CI 0.29–0.38), with an I^2^ value of 87.5%. The pooled 5-year OS in the WBRT + chemotherapy group was 0.55 (95% CI 0.50–0.61), with an I^2^ value of 58.1%. The pooled 5-year OS in the ASCT group was 0.77 (95% CI 0.71–0.84), with an I^2^ value of 0.0%. The pooled 5-year OS in the chemotherapy group was 0.37 (95% CI 0.31–0.42), with an I^2^ value of 88.7%. The results are shown in Fig. 2b.

**Figure 3:**
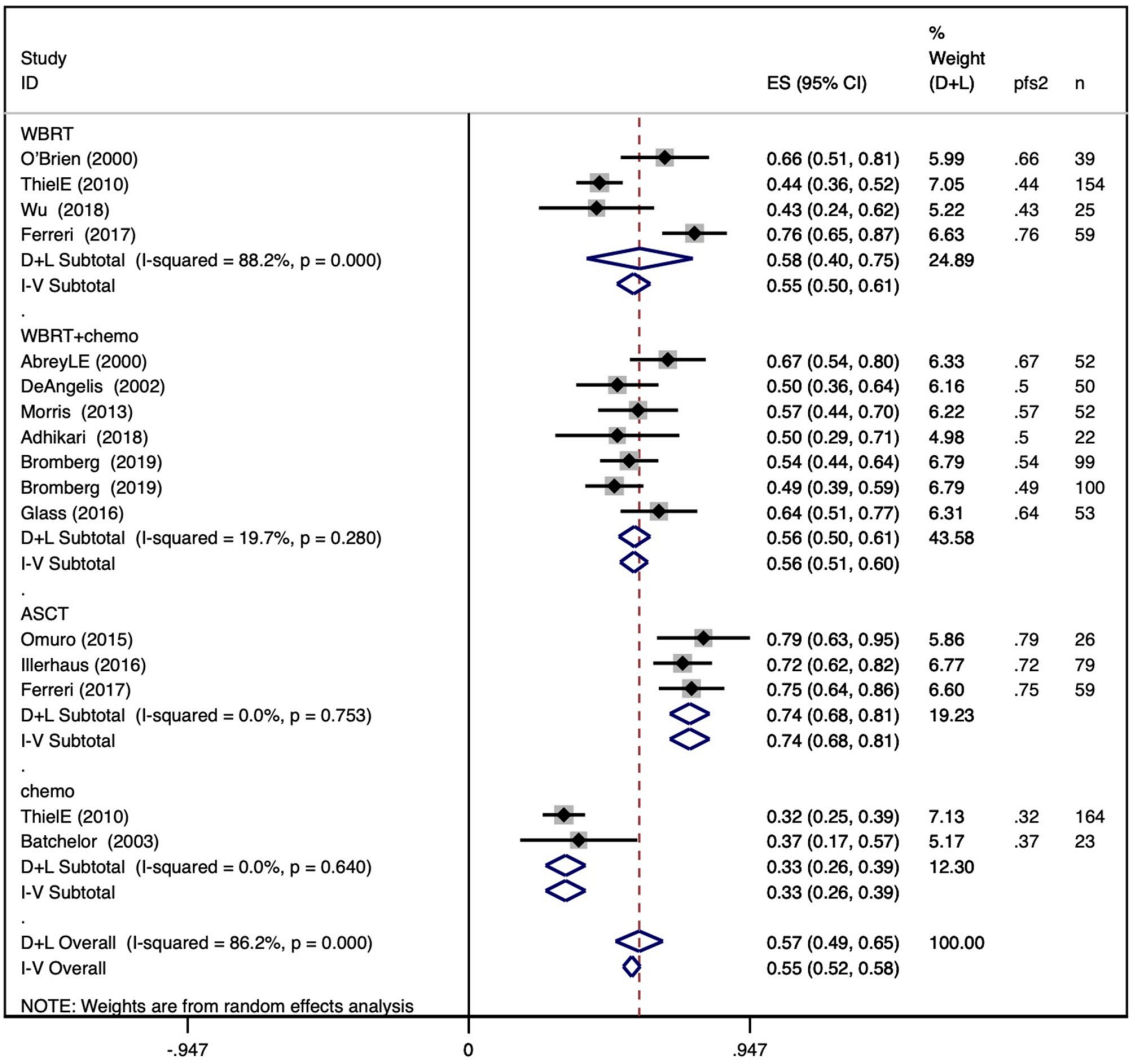

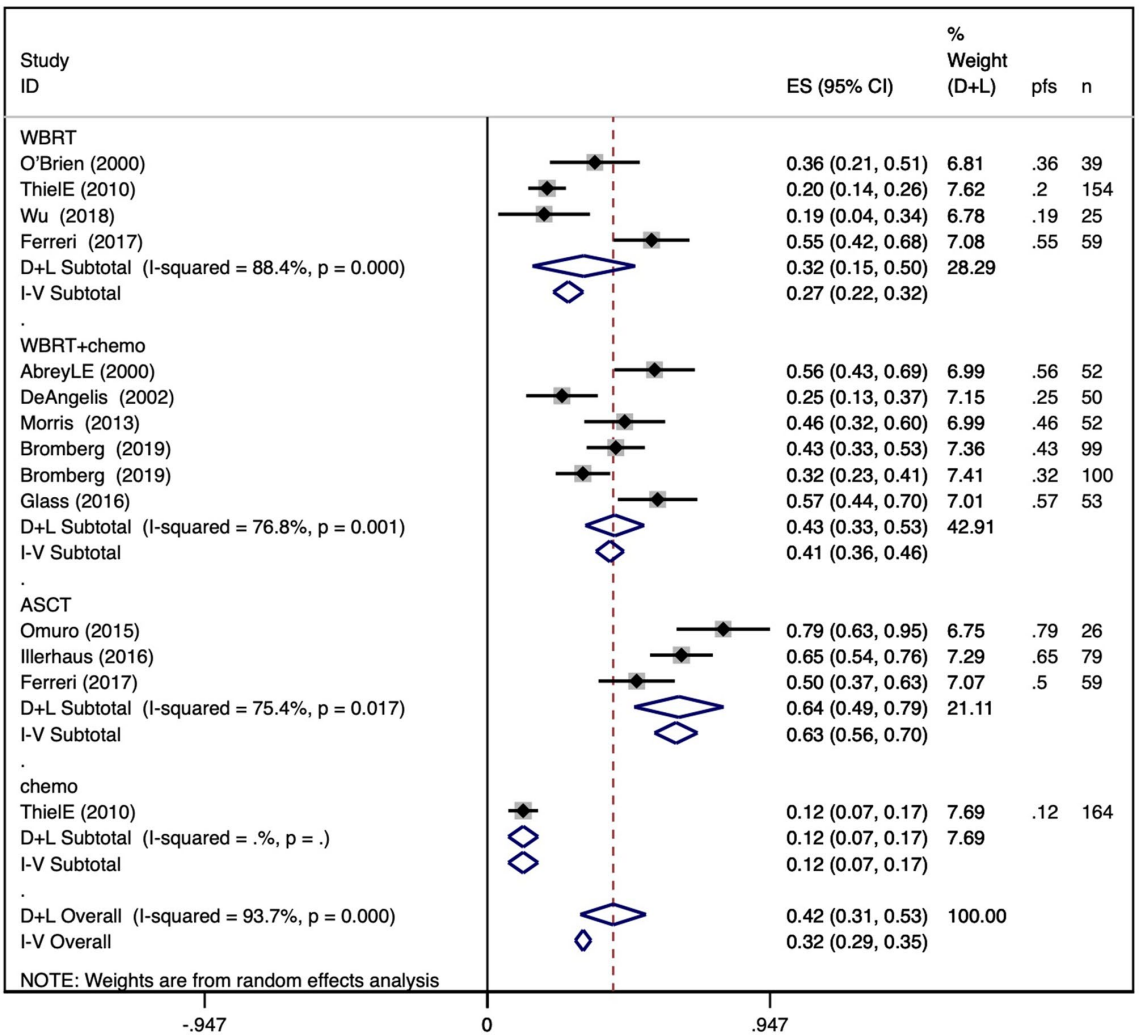
Forest plot of 2-year and 5-year progression-free survival within different consolidation methods. (**a**) Pooled 2-year progression-free survival for WBRT, WBRT + chemotherapy, ASCT, and chemotherapy-only consolidation; (**b**) pooled 5-year progression-free survival for the four consolidation methods.

The pooled 2-year PFS in the WBRT group was 0.55 (95% CI 0.50–0.61), with an I^2^ value of 88.2%. The pooled 2-year PFS in the WBRT + chemotherapy group was 0.56 (95% CI 0.51–0.60), with an I^2^ value of 19.7%. The pooled 2-year PFS in the ASCT group was 0.74 (95% CI 0.68–0.81), with an I^2^ value of 0.0%. The pooled 2-year PFS in the chemotherapy group was 0.33 (95% CI 0.26–0.39), with an I^2^ value of 0.0%. The results are shown in Fig. 3a.

The pooled 5-year PFS in the WBRT group was 0.27 (95% CI 0.22–0.32), with an I^2^ value of 88.4%. The pooled 5-year PFS in the WBRT + chemotherapy group was 0.41 (95% CI 0.36–0.46), with an I^2^ value of 76.8%. The pooled 5-year PFS in the ASCT group was 0.63 (95% CI 0.56–0.70), with an I^2^ value of 75.4%. For 5-year PFS in the chemotherapy group, only one study was included with a result of 0.12 (95% CI 0.07–0.17). The results are shown in Fig. 3b.

### The heterogeneity analysis of the basic characteristics

We conducted univariate meta-regression analyses of basic patient characteristics, such as the proportion of male patients, the median age, median KPS scores, proportion of EGOG with one score or less, multiple sites, DLBCL histology, CSF cytology positivity, CSF protein positivity, and ocular involvement. The results showed that information on these basic characteristics could not explain the source of heterogeneity among the included studies (*P* value all ≥ 0.05). The single dose of MTX in each cycle and the number of cycles given in inductive regimen were also analyzed as factors of heterogeneity for studies included, respectively. The results (β = 0.051, *P* = 0.025, 95% CI 0.007–0.095) showed that the number of cycles given in inductive period could be a source of heterogeneity. Furthermore, as the CR rates may affect the PFS and OS outcomes, we included the best CRR mentioned in each study as one of the covariates for heterogeneity analysis, and the results (β = 0.003, *P* = 0.096, 95% CI − 0.000–0.006) turned out to be of no significant relevance to the heterogeneity of studies within the PFS and OS survival analysis. The results are shown in Table 2.

**Table 2:** Meta-regression analysis of the heterogeneity of the basic characteristics.

Covariates^a^	ES^b^	Coefficient	95% CI	*P* value
Male	24	− 0.418	− 1.152 to 0.316	0.264
Median age	27	0.014	− 0.004 to 0.032	0.116
Median KPS	14	− 0.002	− 0.014 to 0.011	0.811
EGOG ≤ 1	13	− 0.092	− 0.421 to 0.604	0.726
Multiple sites	17	0.085	− 0.369 to 0.539	0.715
DLBCL	18	0.046	− 0.562 to 0.655	0.882
CSF cytology	20	0.236	− 0.214 to 0.685	0.305
CSF protein	10	0.014	− 0.599 to 0.628	0.964
Ocular	22	0.171	− 0.403 to 0.745	0.560
Single dose of MTX (mg/m^2^)	29	− 0.013	− 0.041 to 0.014	0.350
The number of Cycles	29	0.051	0.007 to 0.095	0.025
CR best	28	0.003	− 0.000 to 0.006	0.096

*ES* effect size, *CI* confidence interval.

^a^All of the data in this analysis are presented in Table 1.

^b^Number of studies used in the regression model.

## Discussion

To our knowledge, this is the first systematic review and meta-analysis including almost every prospective clinical trial focused on the first-line treatment of PCNSL patients. First, we compared the complete remission rates (CRRs) of each group classified by the number of drugs in the regimen. When adding another drug to the backbone of HD-MTX, the pooled CCR in the two-drug group was 38% vs. 30% in the mono HD-MTX therapy group. Improved CRRs were also seen in the three- and four-drug groups, with pooled CCRs of 49% and 44%, respectively. Regarding the group administered five drugs or more, whether the addition of more drugs resulted in a better CRR remains unknown, primarily because only two studies with an I^2^ value of 93.4% were included. A combination drug therapy is generally recommended for achieving better CRRs.

We further focused on identifying the optimal drug combination based on HD-MTX. The results of different combinations showed that the MPV regimen (HD-MTX + procarbazine + vincristine) showed the best pooled CRR of 58% and 63% with and without rituximab, respectively. The R-MTX + TMZ (rituximab + HD-MTX + temozolomide) regimen also showed a CRR of 60%, which was comparable to that of the MPV regimen. These results are consistent with the outcome that three- and four-drug regimens have better CRRs.

In an ideal model to compare the survival outcomes of consolidation therapies, the induction methods in each group should be identical. However, when we performed the analysis of the consolidation stage, we found that most studies included in our study did not share the same regimens during the induction and/or consolidation periods; for example, the studies of DeAngelis^16^ and Ferreri^16^ used the same induction therapy but their following consolidation therapies were different.

Therefore, to conduct the comparison, we divided all studies into different groups according to their consolidation methods and compared the effect on the survival outcomes in terms of 2-year and 5-year OS and PFS without taking into consideration their different induction regimens. Meanwhile, a regression analysis was performed to analyse the heterogeneity of all included studies. We incorporated sex, age, performance state, the number of lesions, pathological pattern, CSF or ocular involvement, single dose of MTX, and the number of inductive cycles into the regression analysis. Except for the number of inductive cycles, all other characteristics showed no significant heterogeneity. The best CRRs in each study, which is a factor related to long-term survival, were also included in the regression analysis, but no significant heterogeneity was identified.

We also performed the regression analysis for the survival effect of different consolidation therapies. The results suggested that, in patients who received HD-MTX-based inductive chemotherapy, consolidation treatment with ASCT is associated with the best PFS and OS, followed by the combination treatment of WBRT and chemotherapy. According to the effect of consolidation treatment used WBRT or chemotherapy, WBRT had a better PFS than chemotherapy, while chemotherapy had a much better OS than WBRT.

Our study is a cross-trial comparison study, and has some limitations contributing to the imprecision of the results. Firstly, to some extent, the small size of the included studies may influence the strength of our study. The limited studies are difficult to evaluate the effect of the heterogeneity of the studies, and in our study some results of analyzed I^2^ value equal to zero, which is caused by the the small size of the included studies. Secondly, the high degree of heterogeneity is another limitation of the study. Although the number of chemotherapy cycles given in inductive treatment may partially contribute to the heterogeneity. There are still some other factors that might cause the heterogeneity, such as lymphoma involved the deep areas of CNS or not, meningeal dissemination or not, drug delivery approaches, which were not included in heterogeneity analyse in our study. Thirdly, the selection bias, reporting bias and some other uncertain risks of bias in clinical trials might influence the final results of the meta-analysis. Finally, in our study, we enrolled in the trials using ASCT as consolidation treatment. The trials involving ASCT treatment generally enroll patients with a relatively young age and have fewer comorbidities than patients enrolled in trials without ASCT treatment, which may contribute to the bias of the results. To date, there are few published head-to-head randomized controlled trials in treatments of PCNSL. Our study indirectly compared published prospective single-arm studies. The results could provide a useful information for making choice of the optimal first-line treatment for patients with PCNSL.

## Conclusion

Aside from HD-MTX, there is little consensus on the optimal components of induction and consolidation therapies for newly diagnosed PCNSL. This study provides a comprehensive summary of the available clinical trials for first-line PCNSL treatment. A combination of HD-MTX with other two or three drugs, like MPV regimen with or without rituximab, is likely to offer the highest CRR rates. Besides, with the caveats of a meta-analysis and the trial heterogeneity, studies with ASCT consolidation have been associated with the longest PFS and OS of all analysed subgroups, followed by WBRT combined with chemotherapy. Recently, increased insight into the pathophysiology of PCNSL has led to the introduction of targeted agents into the treatment of PCNSL. We are looking forward to additional randomized clinical trials combining targeted agents with conventional chemotherapy to improve outcomes in the PCNSL population with fewer treatment-associated comorbidities.
